# Transcriptomic data of human adrenocortical NCI-H295R cells treated with cortisol biosynthesis inhibitors^1^

**DOI:** 10.1016/j.dib.2023.109948

**Published:** 2023-12-12

**Authors:** Soo Hyun Kim, Hyun Jung Kim, Jong-Wha Jung, Sooyoung Chung, Gi Hoon Son

**Affiliations:** a Department of Brain and Cognitive Sciences, Scranton College, Ewha Womans University, Seoul 03760, Republic of Korea; b Department of Biomedical Sciences and Department of Anatomy, College of Medicine, Korea University, Seoul 02841, Republic of Korea; c College of Pharmacy, Research Institute of Pharmaceutical Sciences, Kyungpook National University, Daegu 41566, Republic of Korea; d Department of Biomedical Sciences and Department of Legal Medicine, College of Medicine, Korea University, Seoul 02841, Republic of Korea

**Keywords:** Adrenal gland, NCI-H295R, Cortisol, Corticosteroid, Steroidogenesis, Benzimidazolylureas, Metyrapone

## Abstract

Adrenal corticosteroid biosynthesis dysregulation can give rise to various pathological conditions, such as Cushing's syndrome, a disorder characterized by the sustained and excessive production of cortisol. Despite the development of several classes of steroidogenesis inhibitors to treat human diseases associated with cortisol overproduction, their use is limited by insufficient efficacy, adverse effects, and/or tolerability. Recently, we identified a series of benzimidazolylurea derivatives, including the representative compound CJ28, as novel cortisol biosynthesis inhibitors [1]. They significantly inhibited both basal and stimulated production of cortisol in NCI-H295R cells, a human adrenocarcinoma cell line. The inhibitory effects were attributed to both attenuated steroidogenesis and de novo cholesterol biosynthesis. Here, we provide transcriptomic (RNA-seq) data from adrenal cell cultures in response to treatment with either CJ28 or metyrapone (MET), an inhibitor of 11β-hydroxylase). Total RNA was extracted from the cells treated with vehicle (0.1% DMSO), CJ28 (30 µM), or MET (30 µM) for 24 h. Primary sequence data were acquired using paired-end sequencing on an Illumina NovaSeq 6000 platform. The raw RNA-seq data have been deposited in the Gene Expression Omnibus (GEO) database (GSE236435). This dataset is a useful resource for providing valuable information on the gene expression networks underlying adrenocortical steroidogenesis.

Specifications Table

**Table:** 

Subject	Omics: Transcriptomics
Specific subject area	Endocrinology and metabolism, Steroid hormone, Pharmacology
Data format	Raw Analyzed
Type of data	RNA-seq raw data Table Figure/Graph
Data collection	RNA-seq of NCI-H295R cells treated with vehicle (VEH), metyrapone (MET) or CJ28, a recently developed cortisol biosynthesis inhibitor. 1. NCI-H295R cell cultures in serum-free medium were treated with VEH (0.1% DMSO), CJ28 (30 µM) or MET (30 µM) for 24 h. 2. Cells were processed for RNA extraction and then sequenced on Illumina NovaSeq 6000 platform to creating counts files for each transcript in every sample for differential expression analyses.
Data source location	• Institution: Korea University • City/Town/Region: Seoul • Country: Korea, Republic of
Data accessibility	**Repository name: Gene Expression Omnibus** **Data identification number: GSE236435** **Direct URL to data:** https://www.ncbi.nlm.nih.gov/geo/query/acc.cgi?acc=GSE236435
Related research article	S.H. Kim, G.H. Son, S.Y. Seok, S.K. Chun, H. Yun, J. Jang, Y.G. Suh, K. Kim, J.W. Jung, S. Chung. (2023) Identification of a novel class of cortisol biosynthesis inhibitors and its implications in a therapeutic strategy for hypercortisolism, Life Sci. 325:121744. https://doi.org/10.1016/j.lfs.2023.121744

## 1 Value of the Data


 

• These data provide valuable information for understanding the effects of the two cortisol biosynthesis inhibitors with distinct modes of action on genome-wide gene expression profiles in cultured human adrenocortical cells.
• These data can be valuable for researchers interested in studying genes and biological pathways regulating adrenal steroidogenesis. Additionally, they may offer insights into therapeutic strategies for treating pathological conditions linked with excess cortisol production.
• The raw RNA-seq data can be re-analyzed using various workflows and bioinformatics tools, providing additional insights into the molecular mechanisms underlying adrenal corticosteroid biosynthesis.

## 2 Objective

Glucocorticoids (GCs), such as cortisol in primates and corticosterone in rodents, serve as adrenocortical steroid hormones that mediate adaptive responses to stress and coordinate circadian physiology [2]. Hypercortisolism, known as Cushing's syndrome (CS), encompasses pathological conditions characterized by prolonged elevations in circulating GC levels, leading to the development of various metabolic, cardiovascular, immune/inflammatory, or neuropsychiatric diseases [2], [3], [4]. Several steroidogenesis inhibitors, such as metyrapone (MET, an inhibitor of 11β-hydroxylase), ketoconazole (an inhibitor of cytochrome P450-containing enzymes), and mitotane (an inhibitor of 3β-hydroxysteroid dehydrogenase), have been used to treat patients with CS. However, these drugs, that target specific steps of the steroidogenic pathway, still exhibit widespread adverse effects and show unsatisfactory efficacy [3,4]. Recently, we identified a series of benzimidazolylurea derivatives as a novel class of cortisol synthesis inhibitors that simultaneously target both steroidogenesis and de novo cholesterol biosynthesis [1]. Our steroidogenesis inhibitors caused alterations in the mRNA expression of a broad spectrum of genes linked to steroidogenesis, indicating that their actions are evidently distinct from those of the steroidogenic enzyme(s) inhibitors. Therefore, we examined genome-wide RNA expression profiles in cultured human adrenocortical cells in response to treatment with CJ28, a representative benzimidazolylurea derivative, or MET.

## 3 Data Description

**Fig. 1:**
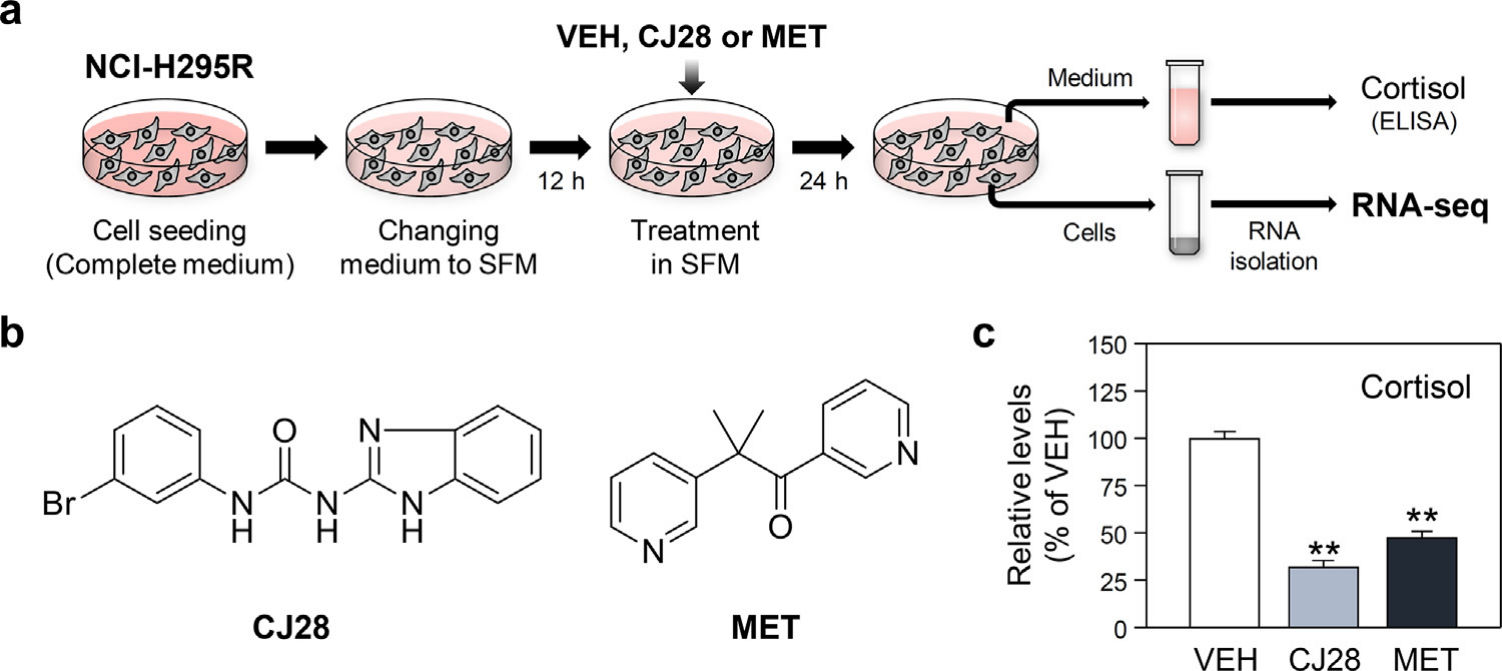
Experimental procedures and validation of the effects of cortisol biosynthesis inhibitors. (a) Schematic representation of experimental procedures. (b) Chemical structures of the cortisol biosynthesis inhibitors used in this study. (c) Inhibitory effects of the compounds on spontaneous cortisol productions from the H295R cell cultures.

We provided RNA-seq data from human adrenocortical NCI-H295R cell cultures treated with two distinct cortisol biosynthesis inhibitors, CJ28 and MET, each with unique chemical structures and modes of action. These treatments were compared with vehicle (VEH: 0.1% DMSO)-treated control cells. The experimental procedures and chemical structures of the cortisol synthesis inhibitors used in the study are shown in Figs. 1a and b, respectively. The inhibitory effects of the chemical compounds were validated by reduced cortisol release into the culture medium (Fig. 1c). The cells used for cortisol measurement were then subjected to RNA-seq experiments as described in the Materials and Methods section. The sequenced library sizes and mapping statistics are summarized in Table 1.

**Table 1:** Key QC metrics of RNA-seq library after alignment with STAR.

Sample	Number of input reads	Averaged mapped length	Uniquely mapped reads (%)	Mismatch rate per base (%)

VEH-1	45,592,694	199.71	92.54	0.23
VEH-2	34,790,181	199.61	92.43	0.23
VEH-3	40,195,909	199.57	92.38	0.23
VEH-4	44,272,933	199.45	92.23	0.24
CJ28-1	41,036,912	200.00	92.12	0.23
CJ28-2	39,579,865	199.67	91.56	0.23
CJ28-3	43,312,876	200.00	91.96	0.23
CJ28-4	33,614,751	199.56	91.48	0.23
MET-1	34,491,960	199.58	92.44	0.23
MET-2	38,060,348	199.60	92.45	0.23
MET-3	37,386,783	199.71	92.54	0.24
MET-4	38,859,163	199.87	92.39	0.23

**Fig. 2:**
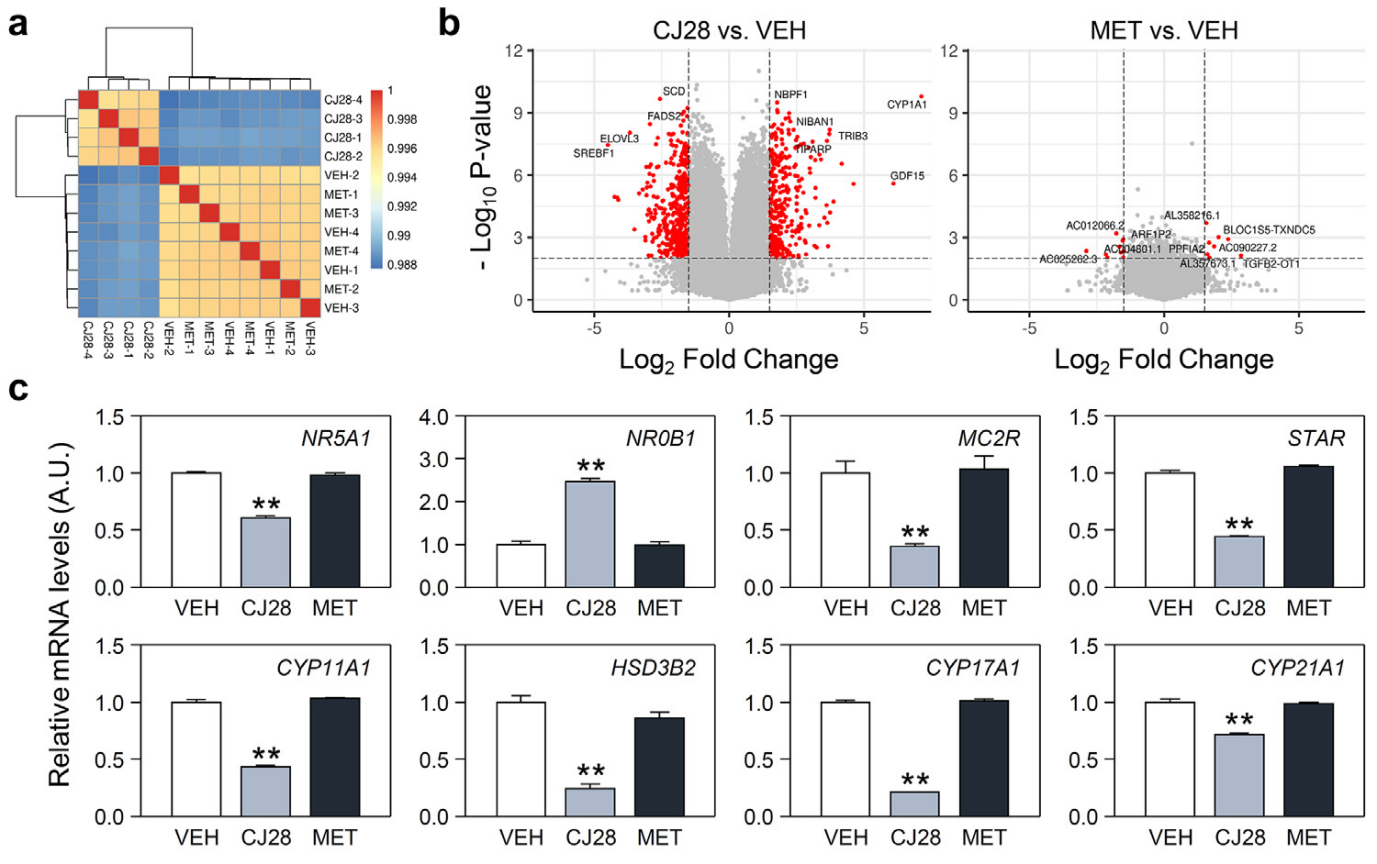
Analyses of DEGs obtained from CJ28- or MET-treated H295R cells. (a) Correlation of gene expression profiles for all pairwise combinations of samples using Pearson's correlation coefficient. (b) Volcano plots showing the DEGs in CJ28- or MET-treated H295R cell cultures. Genes with Log2 fold change (FC) > 1.5 and P < 0.01 were highlighted by red circles. Gene symbols for the top 10 DEGs are indicated. (c) Relative mRNA expression levels of key steroidogenic genes calculated from RNA-seq results. Data are presented as mean ± SEM of arbitrary unit (A.U.), with mean values of VEH group set as 1 (n=4 for each group; **: p<0.01 vs. VEH group by Student's t-test).

Fig. 2a displays the correlation between the gene expression profiles for all pairwise combinations of samples, including CJ28, MET, and VEH. The hierarchical tree indicates that CJ28-treated cells exhibited distinct RNA profiles compared to MET- or VEH-treated cells, which were similar to each other based on normalized gene expression values. The differentially expressed genes (DEGs) after treatment with CJ28 or MET in comparison with the VEH-treated controls are summarized in Fig. 2b. Using the criteria of Log2 fold change (FC) > 1.5 and *P* < 0.01, 787 and 18 genes were identified as DEGs with significantly altered expression by CJ28 and MET, respectively. Fig. 2c exemplifies the relative mRNA expression levels of several key steroidogenic genes, comparing the two cortisol biosynthesis inhibitors. Despite having similar effects on cortisol production, treatment with CJ28 only significantly altered the mRNA expression levels of steroidogenic genes such as *NR5A1, NR0B1, STAR, CYP11A1, HSD3B2, CYP17A1* and *CYP21A1*, whereas MET did not. The DEGs obtained after CJ28 treatment, were utilized for gene enrichment analyses to gain insight into the possible modes of action in the related research article [1]. In this study, we present a comprehensive set of RNA-seq data, including those from MET-treated H295R cells, in addition to previously reported data showing the effects of CJ28.

## 4 Experimental Design, Materials and Methods

### 4.1 Cortisol inhibitors

A benzimidazolylurea derivative, 1-(1H-benzo[d]imidazol-2-yl)-3-(3-bromophenyl)urea (CJ28), was synthesized in-house following our previous report [1]. Metyrapone was purchased from Merck (Burlington, MA, USA).

### 4.2 Cell culture and treatment

NCI-H295R (H295R; ATCC, Manassas, VA, USA) cells were cultured in Dulbecco's modified Eagle's medium-F12 (Thermo Fisher Scientific, Waltham, MA, USA) supplemented with 1% ITS+ Premix (Corning, Corning, NY, USA), 2.5% Nu-Serum (Corning), and 1% antibiotic-antimycotic solution (Thermo Fisher Scientific). The cells were maintained in a humidified incubator containing 5% CO_2_, at 37°C. Confluent cells were incubated for 12 h in serum-free medium and subsequently treated with VEH (0.1% DMSO), CJ28 (30 µM) or MET (30 µM) for 24 h. After the treatment, the culture medium was collected for cortisol measurement. Following this, the cells were washed with cold Dulbecco's phosphate-buffered saline and stored at -80°C until RNA extraction.

### 4.3 Measurement of cortisol production

To validate the inhibitory effects of the inhibitors used in the study, cortisol levels in the culture medium were determined using a commercial enzyme-linked immunosorbent assay (ELISA) kit (Arbor Assays, Ann Arbor, MI, USA). The medium was centrifuged at 4°C for 5 min (10,000 × g) and then subjected to ELISA for cortisol measurement.

### 4.4 Preparation of RNA samples and RNA-seq analysis

Total RNA was isolated from frozen H295R cells using the microRNeasy Mini Kit (Qiagen, Hilden, Germany) following the manufacturer's protocol. RNA integrity was evaluated using an Agilent 2100 Bioanalyzer (Agilent Technologies, Santa Clara, CA, USA), and ribosomal RNA depletion was evaluated using the Ribo-Zero™ reagent (Illumina, San Diego, CA, USA). The cDNA libraries were prepared using the TruSeq™ Stranded Total RNA Prep Kit (Illumina) as per the manufacturer's instructions. Primary sequence data were acquired using paired-end sequencing on an Illumina NovaSeq 6000 platform (Illumina). Raw sequence reads were trimmed for adaptor sequences using Cutadapt software (version 2.8) [5]. The trimmed sequence reads were mapped to GRCh38 using STAR Aligner (version 2.7.10b) [6]. Read count extraction and normalization were carried out using RSEM (version 1.3.3) [7]. The correlation matrix and hierarchical tree were generated to illustrate the similarity among samples using normalized gene expression values obtained from DESeq2 (version 1.38.3) [8]. Differential gene expression was assessed using normalized DESeq2 counts, and volcano plots were visualized using EnhancedVolcano [9].

### 4.5 Data records

The raw reads (fastq) and normalized gene-level read counts in transcript per million (TPM) are available in the NCBI Gene Expression Omnibus (GEO) under accession number GSE236435.

## Limitations

Not applicable.

## Ethics Statement

This study did not involve human participants, animal experiments, or data collected from social media.

## CRediT authorship contribution statement

**Soo Hyun Kim:** Investigation, Writing – review & editing. **Hyun Jung Kim:** Data curation, Visualization, Writing – original draft. **Jong-Wha Jung:** Conceptualization, Resources, Methodology, Visualization, Writing – review & editing. **Sooyoung Chung:** Conceptualization, Supervision, Methodology, Investigation, Writing – original draft, Funding acquisition. **Gi Hoon Son:** Conceptualization, Supervision, Writing – original draft, Funding acquisition.

## Data Availability

Gene expression profiles of human adrenocortical NCI-H295R cells treated with cortisol biosynthesis inhibitors (Original data) (Gene expression omnibus)
